# Understanding the consequence of COVID-19 on undergraduate medical education: Medical students’ perspective

**DOI:** 10.1016/j.amsu.2020.08.045

**Published:** 2020-09-05

**Authors:** Immanuel Sani, Yaser Hamza, Youssef Chedid, Jubilent Amalendran, Nadir Hamza

**Affiliations:** a Leicester Medical School, George Davies Centre, Lancaster Road, Leicester, LE1 7HA, United Kingdom; b GKT School of Medical Education, Kings College London, Great Maze Pond, London, SE1 1UL, United Kingdom; c Medical University of Plovdiv, 4002 Tsentar, Plovdiv, Bulgaria

## Abstract

The coronavirus disease 2019 pandemic has significantly influenced the normal operations of all human affairs on a global scale. Indeed, the pandemic has had a considerable impact on the delivery of medical education in the UK for both pre-clinical and clinical year students. In response to the escalating case fatality rate due to the pandemic, there has been widespread termination of clinical placements, face-to-face teaching sessions, and examinations that require a physical presence by UK medical schools. It is hoped that the cancellation of the aforementioned activities will greatly reduce the exposure of medical students to the coronavirus however, the consequences of these actions may pose substantial issues for the learning experience and professional development of medical students. One such issue is the lack of regular communication between students and personal tutors which may give rise to burnout within students and impede academic performance. Furthermore, the suspension of clinical placements may result in a gradual reduction in students’ clinical skills competence. The practice of medicine is grounded upon the application of basic science which involves undertaking clinical procedures and as such, students may be at a disadvantage due to the missed opportunity to refine these essential skills. In this article, we provide an overview of the consequences of the COVID-19 pandemic on medical education, specifically regarding curriculum delivery and assessment of students. We also adopt a holistic approach by considering the impact of the pandemic on the mental wellbeing of medical students during this unprecedented time. We offer pragmatic suggestions to uphold the quality of medical education such as the implementation of virtual interprofessional education sessions to solve clinical vignettes and virtual consultation skills with simulated patients. We conclude with suggested areas for future research to examine the effectiveness of a virtual interprofessional education model on both short and long-term learning as well as encouraging medical students and academic staff to trial innovative methods of teaching. Despite the resultant complications of the pandemic on medical education, these challenging times may present a serendipitous opportunity for medical students to cultivate the personal attributes expected of a doctor in the face of adversity. In light of the pandemic, there is scope to reconsider the effectiveness of current medical education and welcome innovative methods of delivering education whilst ensuring quality. The combination of recent telecommunication developments with current teaching methodologies may positively change the future landscape of medical education.

## Highlights

• The COVID-19 pandemic has disrupted all stages of undergraduate medical education.
• Students' clinical skills competence may decline as patient access is limited.
• Student support bubbles may reduce the transmission of COVID-19.
• Academic staff and students should trial novel educational measures.

## 1 Introduction

The advent of the coronavirus disease 2019 (COVID-19) pandemic has presented a myriad of adversities worldwide. The resultant disruption in the education of medical students may have a seismic impact on all students at various stages in medical education. The demand for medical schools to train future doctors effectively to ensure the provision of high-quality patient care is rising, yet such interruption to education may result in competency deficiencies within graduates if not acted upon adequately. This article will discuss the consequences of the pandemic on medical education, specifically regarding curriculum delivery and assessment of students, and explore potential solutions to deliver innovative medical education during this unprecedented time.

## 2 Challenges in medical education

Thus far, medical schools have cancelled all face-to-face teaching for students and have subsequently delivered lectures by virtue of utilising online telecommunication platforms. Previous studies have demonstrated that students prefer online teaching modalities to traditional didactic teaching [1]. This is thought to be due to students being able to study more effectively in their home environment and save on travel expenditure which therefore improves attendance [2]. Anecdotally, online lectures have been well-received by students at our respective institutions as students regularly ask lecturers further questions than they would otherwise which in turn facilitates a greater understanding of lecture content. It is important however to note that pre-clinical students may be susceptible to the burnout phenomenon, especially during these extraordinary times where they may have reduced or no contact time with personal tutors for pastoral support [3]. This may also impede a personal tutor's ability to recognise where a student may be facing a biopsychosocial issue.

Medical schools have also suspended clinical placements and observations in hospitals, general practice, and community settings. The effect of this action is two-fold. On one level, students’ clinical skills competence may decline as they no longer have access to patients and simulation models under supervision. Students will, therefore, require costly and time-intensive training upon return to the clinical environment to re-attain the expected level of competency in clinical skills. Additionally, many students in the clinical stage of medical education are unable to complete certain types of research, attend conferences, conduct presentations, and so forth as the nature of these activities will increase COVID-19 transmission if continued. The aforementioned extra-curricular activities are vital when distinguishing candidates that will go on to apply for core or specialty training in the future, therefore the lost opportunities due to the pandemic may induce anxiety over career progression amongst students.

Both written and practical assessments have also been considerably impacted by the pandemic across all medical schools. Many written examinations have been converted to open-book examinations by medical schools in response to the pandemic which raises the potential issue of the examination being an insufficient measure of students’ theoretical knowledge due to ease of access to materials online. To address this issue, the use of online proctoring for all variants of written examinations may be superior to an open-book examination. The presence of an online proctor may simulate a more traditional testing environment whereby students do not have access to resources to answer questions and this will, therefore, necessitate thorough preparation by students to learn relevant theoretical content. Practical assessments have largely been rescheduled to occur in the successive academic year which may result in greater academic burden amongst students as they have to accrue and demonstrate more competencies within a shorter period. Overcoming the interference to practical assessments is more complex and these assessments will likely continue to be postponed until it is appropriate to resume. Medical schools should conscientiously provide ample and protected time for students to prepare for delayed practical assessments to avoid overburdening students.

Current and imminent final-year medical students face the unique issue that follows from the cancellation of traditional Objective Structured Clinical Examinations (OSCEs). OSCEs remain the mainstay of practical assessment at our institutions, and for the first time at our institutions, final-year medical students have had to resit their practical exam virtually. It is probable that virtual examinations will continue to be used until the COVID-19 pandemic recedes. This remains a topic of discussion and uncertainty amongst the incoming final-year medical students as it may confer an unjust disadvantage due to unfamiliarity with a virtual examination.

It is also important to consider how clinical placements will soon be reinstated. As the COVID-19 reproduction (R) number declines over time, social distancing measures will likely be carefully eased. As medical students are reassigned to placements, a certain degree of social distancing may still be in place to reduce student density and COVID-19 transmission. Interestingly, New Zealand has pioneered the use of COVID support bubbles whereby a group of people, who normally have close physical contact, share the same living space. Many student houses will adopt support bubbles which may create local herd immunity and facilitate an easier return to clinical placements. Although, as medical students, we feel that this initiative significantly detracts from the global student experience as we are unable to freely socialise and negatively influence our mental wellbeing. Additionally, support bubbles may be ineffective for vulnerable students and as such, we recommend the development of a risk stratification tool to objectively identify such students and subsequently delay the commencement of their placement until the local R number is deemed to be safe. Furthermore, the limited availability of personal protective equipment for clinicians is well-documented and placement providers need to ascertain if they have PPE capacity to accommodate an influx of students to the healthcare workforce.

Foreign medical electives have also been cancelled and medical students have been limited to local electives based in the United Kingdom. This is typically the main opportunity for medical students to experience global health before the commencement of their post-graduate training. Medical students may, in the future, have to forgo the opportunity to work in a foreign healthcare system or suspend their training to pursue this endeavour.

## 3 Solutions to current challenges

Despite these challenges, the COVID-19 pandemic may provide the impetus for students to exponentially develop their self-directed study skills. Medical school academic staff should actively encourage students to remain resilient and adaptable in the face of the pandemic as this may instil the necessary attributes within students when they transition from student to doctor. The acquisition of such attributes can enable students to manage uncertainty and continue the process of lifelong learning as a doctor more effectively. It is also essential that medical schools provide easily accessible pastoral support for students and schedule regular virtual personal tutor meetings. Greater student support may enhance student learning capacity and academic performance as students gain advice on strategies to better manage academic stress [4].

The postponement of face-to-face consultation skills teaching can be rectified by allowing students to practise virtual consultations with simulated patients in small peer groups. It is well-documented that augmenting doctor-patient communication leads to improved healthcare outcomes. Therefore, the provision of dedicated online consultation teaching could facilitate enhanced student communication skills with patients owing to repetitive practise and instant patient or peer feedback. Currently, at King's College London, we have undertaken virtual tutor groups wherein a clinician serves as the session facilitator and the student presents a patient case to the clinician and 7–11 peers, followed by a reflective discussion on the case and learning points to contemplate. Whilst this experience has been useful to refine the clinical reasoning process, I have gradually perceived these sessions as a monotonous exercise, which is often time-pressured due to the size of the group and the discussions tend to be superficial and less engaging over time. The majority of my peers and I prefer conventional patient bed-side teaching where we can ask patients questions in real-time which therefore allows for a multi-dimensional, heuristic discussion about the patient's care with the educator. Perhaps the implementation of virtual consultations with simulated patients may be an alternative measure to remedy this issue. General practitioners across the UK have successfully utilised telephone and video consultations to manage patient complaints, thus it is therefore logical to suggest that this modality should be paralleled in undergraduate medical education.

As medical students have been withdrawn from clinical placements, there is a risk of a reduction in clinical skills proficiency due to restricted practise. This issue could be addressed by creating projects that require students to actively engage with the task and communicate with peers. From a student perspective, we propose a wider implementation of virtual, group-based interprofessional education (IPE) to discuss and solve clinical vignettes. Multiple studies corroborate the effectiveness of IPE in improving students' understanding of other health profession's competencies and patient crisis management [5]. A novel, virtual model of IPE could potentially achieve similar benefits, and combining such an approach with online education may be widely accepted by medical students.

## 4 Future research implications

We recommend that future research examines both the short and long-term effectiveness of virtual IPE sessions to achieve clearly defined learning outcomes through the utilisation of a standardised assessment measure and the preferred frequency of these sessions amongst medical students. The effectiveness of virtual consultation skills assessment compared to conventional consultation skills assessment also warrants investigation as analogous measures may be implemented for the foreseeable future to constrain COVID-19 transmission.

## 5 Conclusion

These are challenging times. Yet, the COVID-19 pandemic may be opportune to reconsider the effectiveness of current undergraduate medical education and welcome innovative methods of delivering education whilst ensuring quality. The integration of technology will undoubtedly influence current teaching modalities and as such, the outlook of medical education may change forevermore. We hope that this commentary draws attention to potential solutions during this pandemic and encourage students and academic staff alike to trial new educational measures.

## Funding

None to declare.

## Ethical approval

Not required.

## Author contribution

Yaser Hamza – Writing.

Jubilent Amalendran – Writing.

Immanuel Sani – Writing.

Youssef Chedid– Writing.

Nadir Hamza – Writing.

## Guarantor

Immanuel Sani.

## Provenance and peer review

Not commissioned, editor reviewed.

## Declaration of competing interest

None to declare.
